# Prenatal anxiety and the associated factors among Chinese pregnant women during the COVID-19 pandemic——a smartphone questionnaire survey study

**DOI:** 10.1186/s12888-021-03624-1

**Published:** 2021-12-10

**Authors:** Can Cui, Lingling Zhai, Kristin K. Sznajder, Jiana Wang, Xiao Sun, Xiaocai Wang, Weiyu Zhang, Fengzhi Yang, Xiaoshi Yang

**Affiliations:** 1 Department of Social Medicine, College of Health Management, China Medical University, No.77 Puhe Road, Shenyang North New Area, Shenyang, Liaoning Province 110122 P.R. China; 2 Department of Maternal and Child Health, School of Public Health, China Medical University, No.77 Puhe Road, Shenbeixin District, Shenyang, Liaoning Province 110016 P.R. China; 3 Department of Public Health Sciences, College of Medicine, Pennsylvania State University, 90 Hope Drive, Suite 2200, Hershey, PA 17033 USA; 4 Shenyang Women’s and Children’s Hospital, No.41 Shenzhou Road, Shenhe District, Shenyang, Liaoning Province 110000 P.R. China

**Keywords:** Prenatal anxiety, COVID-19, Vomiting, Self-efficacy, Medical care

## Abstract

**Backgrounds:**

The uncertainty of the pandemic of Coronavirus Disease 2019 (COVID-19) brought about tremendous psychological harm for pregnant women, causing their high rates of prenatal anxiety. The impacts of COVID-19 pandemic and symptoms of pregnant status are highly linked with prenatal anxiety. Whereas, self-efficacy and support from family and friends could attenuate the development of prenatal anxiety. Thus, the purpose of the study is to evaluate the prevalence of prenatal anxiety and its influence factors among pregnant women during the pandemic of COVID-19 in Shenyang, China.

**Methods:**

A cross-sectional study with face-to-face interview between April 24, 2020 and May 3, 2020 during the COVID-19 pandemic was applied among pregnant women in Shenyang Women’s and Children’s Hospital. Chi-square tests were calculated to determine the differences in prenatal anxiety among categorical variables. Multivariable logistic regression was employed to investigate the risk factors of prenatal anxiety.

**Results:**

The percentage of prenatal anxiety (GAD-7 score ≥ 7) among pregnant women during the pandemic of COVID-19 was 34/304 (11.18%). Logistic regression indicated that vomiting (OR 4.454, 95% CI 1.113–17.821) and feeling susceptible to SARS-CoV-2 infection (OR 2.966, 95% CI 1.151–7.642) increased the odds of prenatal anxiety. Satisfaction with medical care (OR 0.303, 95% CI 0.113–0.813) and self-efficacy (OR 0.253, 95% CI 0.100–0.639) decreased the odds of prenatal anxiety. High monthly income (OR 0.246, 95% CI 0.078 ~ 0.780) reduced the chances of suffering from prenatal anxiety.

**Conclusion:**

The pregnant women in China exerted a higher prevalence of prenatal anxiety during the COVID-19 pandemic than that without COVID-19 pandemic. Effective management on symptoms of pregnant status should be delivered to relieve prenatal anxiety for the pregnant women. Furthermore, interventions on self-efficacy enhancement and high-quality medical prenatal care should be provided to prevent from the susceptibility of SARS-CoV-2 infection and reduce prenatal anxiety.

## Background

Pregnant women experience considerable physical and psychological changes in the process of pregnancy, which may elevate their likelihood of suffering from mental disorders [1, 2]. Since December 2019, pregnant women have had to experience the Coronavirus disease 2019 (COVID-19) outbreak [3], which may detrimentally impact their psychological health. The uncertainty of the COVID-19 pandemic brought about not only the risk of morbidity and mortality caused by COVID-19, but also resulted in mental disorders among pregnant women, such as symptoms of anxiety, panic, depression, and post-traumatic stress disorder (PTSD) [4–6].

Pregnant women may cancel or delay obstetric examinations because of worries about the risk of SARS-CoV-2 infection at health facilities, routine inspection and the process of delivery [7, 8]. The fear of infection brought about excessive burdens for women and presumably exacerbated symptoms of anxiety which has been found to be prevalent in pregnancy [9]. According to the previous studies, approximately 8.3–57.0% of pregnancy suffered from high anxiety prevalence during the COVID-19 pandemic [10–13]. Anxiety may have inimical consequences on the outcomes of pregnancy, increasing the risks of preeclampsia, nausea and vomiting, resulting in preterm labor or miscarriage and reduced quality of life [14, 15]. Furthermore, it may also result in adverse effects on the newborns including low birth weight, negative infant temperaments, and adverse neurodevelopmental outcomes [16].

Factors that influence pregnant women’s psychological state are complicated and multifaceted. Demographic characteristics and characteristics of pregnancy such as monthly income and vomiting were found to be associated with prenatal anxiety [8, 17]. Pregnant women who have had financial strain and suffered from vomiting, especially hyperemesis gravidarum, reported a deterioration of their physical and mental health, which increased their perception of anxiety [18, 19]. Besides the well-documented perinatal risk factors, social cognitive model indicated that general public’s assessment of cognitive processes and emergencies directly affect their emotional responses [20]. Based on the social cognitive model, worry about SARS-CoV-2 infection may be a crucial predictor associated with increased anxiety rates in pregnant women [21]. Meanwhile, the conservation of resources theory suggests that restoration of resources, such as medical care, can mitigate the consequences of large-scale traumatic events and has been shown to be the most influential predictor in reducing post-traumatic stress [22].

Self-efficacy, as a positive resource, is defined as the perceptual ability to execute and perform required actions in order to achieve a specified type of performance, which plays a vital role in the transition into motherhood and has a strong relationship with reduced anxiety [23, 24]. As the motivation of individual self-continuous adjustment and a reflection of a positive state of mind, self-efficacy functions as a vital part in the transition into the role of motherhood and the reduction of anxiety [25]. Pregnant women who have high levels of self-efficacy can more easily confront psychological pressure and actively seek effective ways to solve problems when they encounter challenges. A growing body of research illustrates that self-efficacy acts as a buffer to daily stress, which contributes to greater psychological well-being and physical health [26, 27].

There is an urgent need to explore the mental health among pregnant women during COVID-19 pandemic. The target of this study was to assess the levels of prenatal anxiety and its associated protective and risk factors during the COVIID-19 pandemic, including demographic characteristics, symptoms of pregnancy status, and positive resource, as well as the pandemic of COVID-19. This study provides evidence that can be used to improve psychological counseling and psychological crisis interventions for pregnant women.

## Materials and methods

### Study design

A cross-sectional study with face-to-face interview between April 24, 2020 and May 3, 2020 during the COVID-19 pandemic was measured among pregnant women in Shenyang Women’s and Children’s Hospital, Liaoning province, China. Pregnant women who came for routine obstetric examinations or hospitalizations, met the inclusion criteria, were continuous selected in this study. This study was implemented and approved by the Standards of Ethics Committee at China Medical University. Questionnaires were set up in advance before the survey and the questionnaire was preset, only after all questions have been answered by the respondents that could be submitted. The questionnaire included the Generalized Anxiety Disorder 7-item Scale (GAD-7), General Self-Efficacy Scale (GSES), questions on demographic characteristics, questions on symptoms of pregnant status, and questions on impacts of COVID-19 response, taking about 25 min to complete.

### Participants

The inclusion criteria for the participants was: I. Eighteen years and older; II. Visited the hospital and had maternal health records available between April and May 2020; III. Able to use a mobile phone and complete the questionnaire independently or with the help of the interviewer; IV. Willing to participate and provide signed web-based informed consent. Study exclusion criteria included: I. Reported a hearing or vision impairment, mental illness, or clinical diagnosed psychiatric disorder including anxiety, depression, mania, schizophrenia.; II. Reported fetal malformations, stillbirths and other fetal abnormalities requiring the induction of labor.

A comprehensive explanation of this survey purpose, content, was provided by experienced surveyors in advance of this questionnaire, and then pregnant women who prefer to participate in this study were required to sign an online informed consent forms, data were attained through trained surveyors with face-to-face interview through a link to an electronic version of questionnaire on a WeChat platform via Wenjuanxing Platform, a widely used social media and professional online survey platform in China. Among totally 355 pregnant women identified, 304 consecutive pregnant women validly completed questionnaires, obtaining a valid response rate of 83.3%.

### Instruments

#### Demographic characteristics of the pregnant women and characteristics of pregnancy

Demographic characteristics of the participants including age (18–29, 30–34, ≥35), education (junior college and below, bachelor’s degree and above), monthly income (≤3000yuan, 3001-5000yuan, >5000yuan), trimester of gestation [first and second trimester (gestational period ≤27 + 6 weeks), third trimester (≥28 weeks)], history of abortion (yes/no), were collected in this study.

#### Symptoms of pregnant status

Symptoms of pregnant status included gravidity (first pregnancy/two or more previous pregnancies), pregnancy complications (yes/no), vomiting (ever/never), mood fluctuations (yes/no).

### Measurement of self-efficacy

Self-efficacy was conducted with the assessment using the Schwarzer General Self-Efficacy Scale (GSES) [28]. The Chinese version of GSES was developed by Wang Ck, is widely used for population research, and has good reliability and validity [29]. The GSES scale is comprised of 10 items and each item is answered on a 4-point Likert scale, which includes “not at all true” (1), “hardly true” (2), “moderately true” (3), and “exactly true” (4). Scores of all the items were summed. The overall higher score shows a greater levels of self-efficacy. The cut-off score of the self-efficacy was 20, which was the average level of the participants. In this study, the Cronbach’s alpha for the GSES scale was 0.962.

### Measurement of the impacts of COVID-19

Measures of COVID-19 worries regarding the COVID-19 pandemic include the questions on whether or not they felt large impacts on daily life during the COVID-19 pandemic (yes/no), whether or not they felt susceptible to SARS-CoV-2 infection (yes/no), whether or not communities where they lived were under lockdown during the COVID-19 pandemic (yes/no), whether or not they were satisfied with their medical care (yes/no),and timely support from family and friends (yes/no).

### Measurement of prenatal anxiety

Prenatal anxiety during pregnancy was measured by the Generalized Anxiety Disorder 7-item Scale (GAD-7) [30]. The Chinese version of GAD-7 consisting of seven items was developed by Gong Y and has good reliability and validity [31]. The GAD-7 is answered by the 4-point Likert from 0 (not at all) to 3 (nearly every day). In this study, the cut-off score of GAD-7 recommended for the population of women with pregnancy was 7 according to previous research [32]. Cronbach’s alpha of this scale was 0.878, exhibiting excellent reliability. In addition, the average variance Extracted (AVE) of GAD-7 are 0.584 in this study, indicating good validity.

### Statistical analysis

Chi-square tests were employed for the categorical variables to compare the differences in the prevalence of prenatal anxiety. Multivariable logistic regression was employed to investigate the associated factors of prenatal anxiety. The demographic characteristics and characteristics of pregnancy, symptoms of pregnant status as well as impact of COVID-19 were selected as the independent variable. Anxiety were converted into categorical variables as a dependent variable in this study based on the research purpose. *P* values < 0.05 were thought as statistically significant for the study. Odds ratios (OR) and 95% confidence levels (95% CI) were calculated to analyze risk factors associated with anxiety. All analyses were employed with SPSS 24.0 statistical software version 3 for Windows version 17.0 (SPSS,Inc., Chicago, IL).

## Results

### Characteristics of pregnant women and distributions of prenatal anxiety

A total of 304 women with pregnancy completed this online survey. The demographic characteristics and the prevalence rate of prenatal anxiety in pregnant women are exhibited in Table 1. The percentage of prenatal anxiety among pregnant women was 34/304 (11.18%). The age of was 30.07 (SD = 3.68) on the average. Half of the pregnant women reported a bachelor’s degree or above (52%). Few women (18.4%) had a monthly income lower than 3000–5000 yuan. The majority of participants were in their third trimester (76.6%). For most subjects (63.2%), it was their first pregnancy and 29.6% of participants had a history of abortion. About one-third of women had pregnancy complications (35.9%), vomiting (22.4%), or mood fluctuations (34.2%). The majority of pregnant women reported high levels of satisfaction with their medical care (85.5%) and had a high sense of self-efficacy (80.3%).

**Table 1:** Demographic characteristics and factors related to prenatal anxiety among pregnant women during the COVID-19 pandemic in Shenyang China (*N* = 304)

Variables	Number(%)	Non-Prenatal Anxiety (%)	Prenatal Anxiety(%)	X^**2**^	***P***
**Demographic characteristics and characteristics of pregnancy**					
**Age (years)**					
18–29	151(49.7%)	129(85.4%)	22(14.6%)	4.517	0.100
30–34	118(38.8%)	107(90.7%)	11(9.3%)		
≥ 35	35(11.5%)	34(97.1%)	1(2.9%)		
**Education**					
Junior college and below	146(48.0%)	133(91.1%)	13(8.9%)	1.470	0.225
Bachelor’s degree and above	158(52.0%)	137(86.7%)	21(13.3%)		
**Monthly income (CNY)**					
≤ 3000	56(18.4%)	44(78.6%)	12(21.4%)	7.392	0.025*
3001–5000	136(44.7%)	123(90.4)	13(9.6%)		
> 5000	112(36.8%)	103(92.0)	9(8.0%)		
**Trimester of gestation**					
First and second trimester	71(23.4%)	65(91.5%)	6(8.5%)	0.697	0.404
Third trimester	233(76.6%)	205(88%)	28(12%)		
**History of abortion**					
Yes	90(29.6%)	83(92.2%)	7(7.8%)	1.494	0.222
No	214(70.4%)	187(87.4%)	27(12.6%)		
**Symptoms of pregnant status**					
**Gravidity**					
1 time	192(63.2%)	165(85.9%)	27(14.1%)	4.346	0.037*
≥ 2 times	112(36.8%)	105(93.8%)	7(6.3%)		
**Pregnancy complication**					
Yes	109(35.9%)	97(89.0%)	12(11.0%)	0.005	0.942
No	195(64.1%)	173(88.7%)	22(11.3%)		
**Vomiting**					
Ever	68(22.4%)	63(92.6%)	5(7.4%)	1.294	0.025*
Never	236(77.6%)	207(87.7%)	29(12.3%)		
**Mood fluctuations**					
Yes	104(34.2%)	91(87.5%)	13(12.5%)	0.276	0.040*
No	200(65.8%)	179(89.5%)	21(10.5%)		
**Impacts of COVID-19**					
**Reported large impact on daily life during the COVID-19 pandemic**					
Yes	193(63.5%)	168(87.0%)	25(13.0%)	1.666	0.197
No	111(36.5%)	102(91.9%)	9(8.1%)		
**Perceived susceptibility to SARS-CoV-2 infection**					
Impossible	134(44.1%)	125(96.3%)	9(6.7%)	7.785	0.028*
Somewhat possible	129(42.4%)	107(82.9%)	22(17.1%)		
Very possible	41(13.5%)	38(92.7%)	3(7.3%)		
**Community in lockdown**					
Yes	236(77.6%)	208(88.1%)	28(11.9%)	0.419	0.483
No	68(22.4%)	62(91.2%)	6(8.8%)		
**Satisfaction with medical care**					
Yes	260(85.5%)	237(91.2%)	23(8.8%)	9.886	0.002**
No	44(14.5%)	33(75%)	11(25%)		
**Self-efficacy**					
≤ 20	60(19.7%)	47(78.3%)	13(21.7%)	8.269	0.004**
> 20	244(80.3%)	233(91.4%)	21(8.6%)		
**Timely support from family and friends**					
Yes	244(80.3%)	221(90.6%)	23(9.4%)	3.846	0.050
No	60(19.7%)	49(81.7%)	11(18.3%)		

*CNY* Chinese yuan

*Significant at the 0.05 level (two-tailed)

**Significant at the 0.01 level (two-tailed)

The factors associated with prenatal anxiety during the COVID-19 pandemic are represented in Table 1. Women with a monthly income of less than 3000 yuan were prone to experience anxiety compared with other groups (*P* < 0.05). Primigravid women reported a higher rate of anxiety compared with those who had two or more previous pregnancies (*P* < 0.05). Pregnant women with vomiting symptoms experienced a higher prevalence of anxiety than those without vomiting symptoms (*P* < 0.05). Pregnant women who felt susceptible to SARS-CoV-2 infection suffered a higher percentage of anxiety than those who felt they could not possibly be infected with SARS-CoV-2 (*P* < 0.01). Participants who were dissatisfied with medical care had higher pregnancy-related anxiety compared with participates who were satisfied with their medical care (*P* < 0.01). Pregnant women enjoying with more self-efficacy had a higher prevalence of anxiety compared with other groups (*P* < 0.01).

### The risk factors of prenatal anxiety

The associated factors of prenatal anxiety was analyzed with multivariable logistic regression analyses. High monthly income (OR 0.246, 95% CI 0.078 ~ 0.780) and (OR 0.289, 95% CI 0.101 ~ 0.822), satisfaction with medical care (OR 0.303, 95% CI 0.113 ~ 0.813), and self-efficacy (OR 0.253 95% CI 0.100 ~ 0.639) decreased the chances of suffering from prenatal anxiety. Conversely, vomiting (OR 4.454, 95% CI 1.113 ~ 17.821) and felt perceived susceptible to SARS-CoV-2 infection (OR 2.966, 95% CI 1.151 ~ 7.642) increased the odds of prenatal anxiety.

**Fig. 1:**
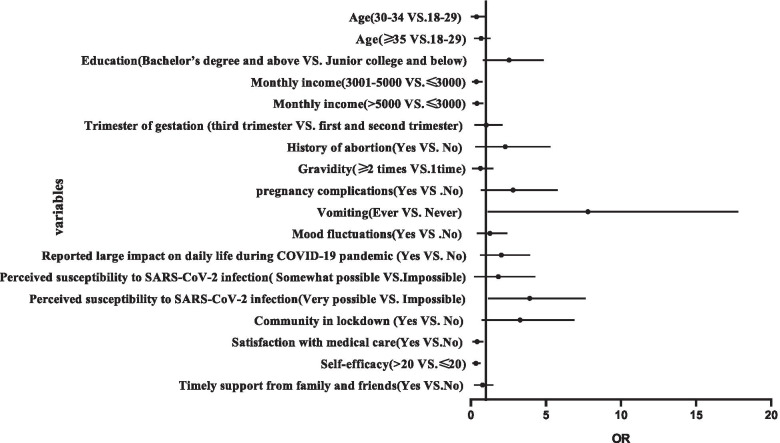
Forest plot of the risk factors for anxiety (Multiple logistic regression). Abbreviations: OR, odds ratio

**Fig. 2:**
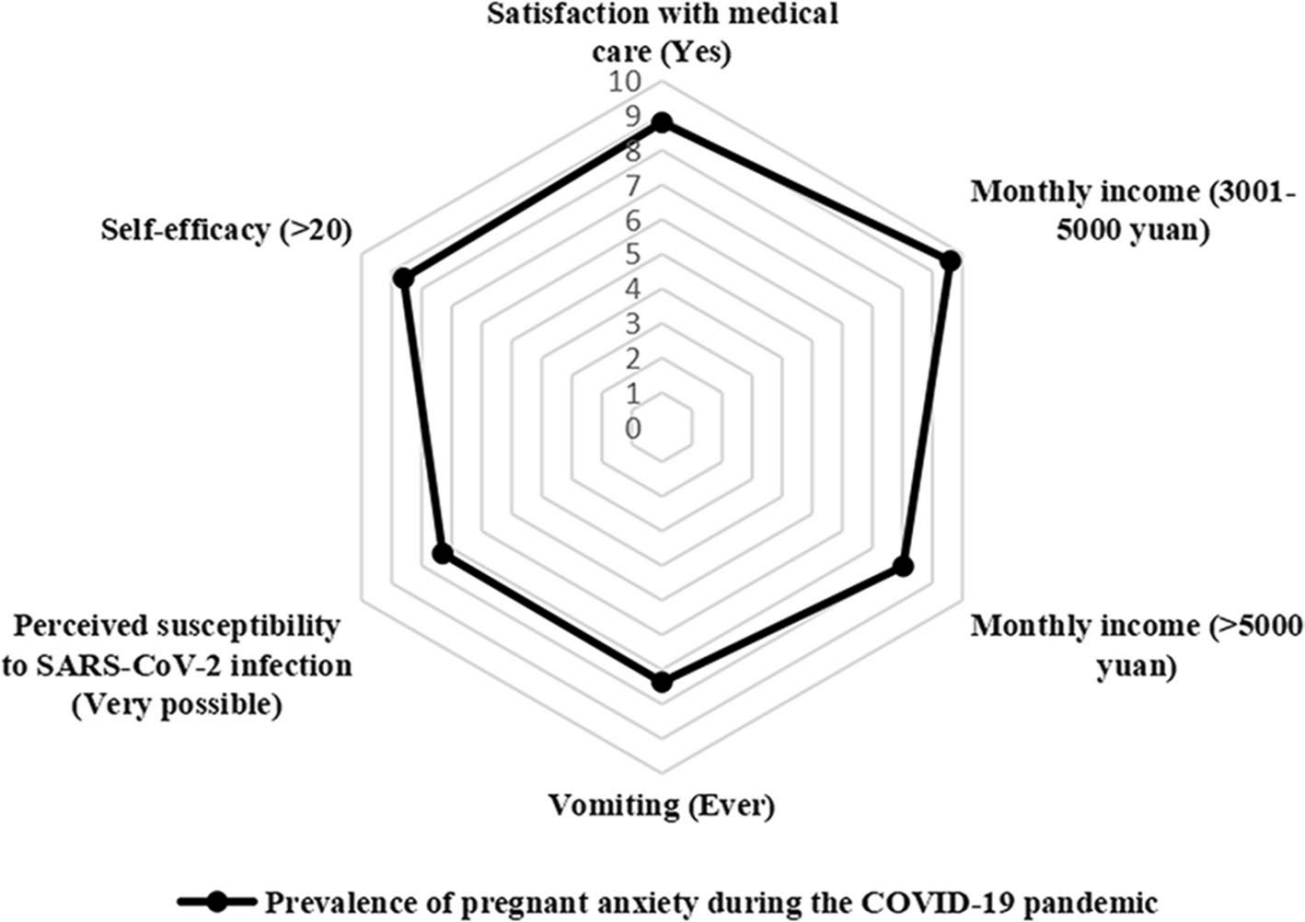
Radar chart of anxiety risk factors

The results of the final multivariable logistic regression model and the forest plot are listed in Fig. 1 and Table 2, respectively. The radar chart of the risk factors of anxiety is shown in Fig. 2. High monthly income, satisfaction with medical care, and high self-efficacy were independent protective factors of prenatal anxiety. Conversely, vomiting and feeling susceptible to SARS-CoV-2 infection were risk factors of prenatal anxiety.

**Table 2:** The multivariable logistic regression analysis for exploring factors of prenatal anxiety during the COVID-19 pandemic

Variables	Odds ratio	95%***CI***
**Demographic characteristics and characteristics of pregnancy**		
**Age (years)**		
18–29	REF	
30–34	0.098	0.009–1.010
≥ 35	0.521	0.208–1.305
**Education**		
Junior college and below	REF	
Bachelor’s degree and above	1.970	0.799–4.858
**Monthly income (CNY)**		
≤ 3000	REF	
3001–5000	0.246	0.078–0.780
> 5000	0.289	0.101–0.822
**Trimester of gestation**		
Second trimester	REF	
Third trimester	0.706	0.238–2.098
**History of abortion**		
No	REF	
Yes	1.256	0.297–5.306
**Symptoms of pregnant status**		
**Gravidity**		
1 time	REF	
≥ 2times	0.355	0.085–1.482
**Pregnancy complications**		
No	REF	
Yes	1.979	0.678–5.778
**Vomiting**		
Never	REF	
Ever	4.454	1.113–17.821
**Mood fluctuations**		
No	REF	
Yes	0.983	0.398–2.432
**Impacts of COVID-19**		
**Reported large impact on daily life during the COVID-19 pandemic**		
No	REF	
Yes	1.548	0.610–3.931
**Perceived susceptibility to SARS-CoV-2 infection**		
Impossible	REF	
Somewhat possible	0.968	0.219–4.287
Very possible	2.966	1.151–7.642
**Community in lockdown**		
No	REF	
Yes	2.219	0.715–6.888
**Satisfaction with medical care**		
No	REF	
Yes	0.303	0.113–0.813
**Self-efficacy**		
≤ 20	REF	
> 20	0.253	0.100–0.639
**Timely support from family and friends**		
No	REF	
Yes	0.581	0.224–1.506

*95% CI* 95% confidence interval, *REF* reference group, *CNY* Chinese yuan

## Discussion

The study demonstrated the impacts of the COVID-19 pandemic on the prevalence of prenatal anxiety among 304 pregnant women in Shenyang, the capital city of Liaoning province, China. The results revealed that 11.18% pregnant women experienced prenatal anxiety during the COVID-19 pandemic (GAD score ≥ 7), which was slightly higher than the prevalence before the pandemic according to the previous studies (4% VS 7%) [33, 34]. Previous surveys also indicated that the degree of anxiety in the presence of the COVID-19 pandemic among pregnant women in Shenyang was slightly lower than that of those who lived in Wuhan (24.5%), U.S. (22.7%), Canadian (36.2%), Sri Lanka (17.5%), but was higher than those who lived in Chongqing (10.4%) [6, 10, 35, 36]. The possible reasons might be due to Shenyang being in a low-risk area for SARS-CoV-2 [37]. Additionally, the Chinese government provided timely information and conducted universal education on prevention and control measures to enable the public to correctly understand the COVID-19 pandemic and its transmission route [38]. Various hospitals provided convenient timely medical access for pregnant women, including reduced waiting times for prenatal care which also lessened crowding at medical facilities [39], which might make pregnant woman feel more secure and thus, decrease the symptoms of anxiety. In addition, this study conducted from April to May 2020, when the epidemic prevention and control in China had achieved national recognition, which may have helped alleviate pandemic-related fear for the general public.

This study revealed that high monthly income was a protective factor of pregnant women’s prenatal anxiety, which was consistent with previous studies [40]. Having a higher monthly income might result in a reduced impact of the COVID-19 pandemic on the women’s daily life and could facilitate access to higher-quality medical resources [18]. Additionally, pregnant women with low monthly income may not receive adequate attention from their families due to financial deprivation, resulting in relatively low access to health services and even a lack of prenatal care during the COVID19 pandemic, which might trigger anxiety [35].

Notably, vomiting was found to be a risk factor for prenatal anxiety. Women with a history of severe vomiting were susceptible to have psychological disturbances, such as anxiety and depression [41]. Vomiting may act as a physiological response to overwhelming stress and therefore pregnant women with vomiting symptoms may be more likely to experience anxiety when they were not able to cope effectively with stress caused by the epidemic of COVID-19 [42]. Pregnant women who have less pregnancy experience and more sensitive may easily magnify pregnancy symptoms such as nausea, vomiting into a more serious illness, or even be associated with COVID-19 infection, which finally enhance prevalence of anxiety. This finding was consistent with a prospective case-control study, which demonstrated that the degree of anxiety in women with pregnancy with symptoms of vomiting was higher than that among non-vomiting pregnant women when suffered from elevated stress [41]. Thus, vomiting symptoms should be monitored as a regular obstetric examination. High-quality prenatal care and long-term follow-up should be provided for the pregnant women with pregnancy-related complications to promote early diagnosis and attenuate the levels of anxiety during the COVID-19 pandemic.

The results of this study indicated that pregnant women who perceived susceptible to SARS-CoV-2 infection were prone to have higher levels of prenatal anxiety. Among our participants, 13.5% of women reported feeling very possible about SARS-CoV-2 infection. Given the common modalities of routine examination, pregnant women were afraid of being exposed to hospitals where there were relatively high cases of COVID-19 patients. Besides, with the uncertain features of the COVID-19 virus and symptoms, the excessive concerns and worries about their own health and vertical mother-to-fetus transmission infection might have increased the prevalence of anxiety [21]. In addition, fear of discrimination infectious diseases increases people’s concern for infection and drives adverse psychological impacts, which could finally result in elevated levels of anxiety [43]. This finding was consistent with a study of 2740 American prenatal women form 47 states and a survey of 4142 prenatal women from 10 provinces in China, which both demonstrated that the more worried about COVID-19 pandemic, the more severe their anxiety would become [17, 44]. Thus, appropriate intervention on mental health management through disseminating accurate information and providing health guidance is crucial to alleviate the risk of inaccurate perception among pregnant women and prevent from the development of anxiety during the COVID-19 pandemic.

Another finding of our survey was that satisfaction with medical services serves as a protective factor against prenatal anxiety. The majority of pregnant women (260/304, 85.5%) reported they were satisfied with medical services, which implied that the quality of prenatal care improved and the expectations of pregnant women’s health were met during the COVID-19 pandemic. Chinese pregnant women were satisfied with local health facilities due to the effective measures to facilitate prenatal care, such as prompted interpreted the guidelines through new media to popularized professional knowledge, launched online education to answer questions, opened online appointments and green channels [39]. Pregnant women who were satisfied with their medical care tended to report experiencing warmth, security, and easier access to medical services, which might attenuate the impact of potentially adverse psychological effects due to the COVID-19 pandemic, such as anxiety. Our finding was consistent with a survey including 100 cancer patients showing that satisfaction of medical care not only influenced the compliance of patients and reduced disease-related stress and the prevalence of anxiety, but also impact the treatment outcomes [45]. Therefore, this research provides evidence-based evidence that all pregnant women should be provided with access to high-quality prenatal care during the pregnancy.

Finally, our findings illustrated that self-efficacy played a positive role in relieving anxiety, which is consistent with previous studies [25, 27]. Self-efficacy can buffer the effects of stressful life events by enhancing the ability to combat the stressors [46] . Pregnant women were susceptible to perceive additional stress in the COVID-19 pandemic. High degrees of self-efficacy might help pregnant women seek social support from their family, friends, and physicians, which finally recover quickly from the detrimental influence of the COVID-19 pandemic [47, 48]. Previous study also found that enhance the level of self-efficacy among pregnant women were prone to promoting mental well-being and combating psychological distress during the COVID-19 pandemic [17]. Therefore, psychological interventions promoting self-efficacy should be provided for pregnant women to enhance their confidence during the process of pregnancy and reduce their anxiety.

### Limitations

There are several limitations for the present study. First, this study’s cross-sectional design indicating that it is impossible to draw conclusions about the causal relationships between anxiety and its associated factors. Therefore, prospective longitudinal studies are needed to confirm the results presented in this study. Second, participants in this study were only from a single hospital in an urban area in northeastern China, which does not allow for the generalizability of this results to other populations with selection bias and information bias. Therefore, expanding hospitals and regions to improve the results of generalizability should be prominent in the future study. Third, this survey did not take account of the influence of individual factors (such as social media usage, occupation), obstetric characteristics (fear of giving birth, planned pregnancy) on pregnant women’s anxiety, which could be confounding factors [49–52]. Therefore, future study should be further considered to explore the influence of these factors on pregnant women’s anxiety during the COVID-19 pandemic.

## Conclusion

Our study revealed that there is a slightly high rate of prenatal anxiety among Chinese pregnant women in COVID-19 pandemic (11.18%). Pregnant women who suffered from vomiting and perceived susceptible to SARS-CoV-2 infection experienced the elevated levels of prenatal anxiety. Moreover, high monthly income, satisfaction with medical care, and self-efficacy were found to be protective factors against prenatal anxiety for pregnant women. This study indicated that interventions aiming to target reactions including improvement of self-efficacy, provision of high quality prenatal care and management of pregnancy-related complications are of significant importance to alleviate anxiety among pregnant women while they are encountering public health emergencies.

## Data Availability

The datasets generated and/or analysed during the current study are not publicly available because this study was a minor study of a project which focus on mental health of public during the COVID-19 pandemic. But the datasets are available from the corresponding author on reasonable request.

## Abbreviations

COVID-19: Coronavirus disease 2019
CI: Confidence interval
GSES: General Self-Efficacy Scale
GAD-7: Generalized Anxiety Disorder 7-item Scale
OR: odds ratio
CNY: Chinese yuan
REF: Reference group
